# The resident macrophages in murine pancreatic islets are constantly probing their local environment, capturing beta cell granules and blood particles

**DOI:** 10.1007/s00125-018-4592-4

**Published:** 2018-03-27

**Authors:** Bernd H. Zinselmeyer, Anthony N. Vomund, Brian T. Saunders, Michael W. Johnson, Javier A. Carrero, Emil R. Unanue

**Affiliations:** Department of Pathology and Immunology, Division of Immunobiology, Washington University School of Medicine, BJC Institute of Health, Campus Box 8118, 660 S. Euclid Avenue, St. Louis, MO 63110 USA

**Keywords:** Beta cells, Fluorescence microscopy, Islet, Islet autoimmunity, Macrophage

## Abstract

**Aims/hypothesis:**

We studied here the interactions between the resident macrophages of pancreatic islets with beta cells and the blood vasculature. We also examined the immunological consequences of such interactions.

**Methods:**

Islets were isolated from C57BL/6 mice expressing CX3C motif chemokine receptor 1–green fluorescent protein (CX3CR-GFP) and examined live by two-photon microscopy. Islets were also examined by electron microscopy to study the relationship of the intra-islet macrophages with the beta cells. In NOD.*Rag1*^−/−^ mice and young (non-diabetic) male mice, the acquisition of beta cell granules was tested functionally by probing with CD4^+^ T cells directed against insulin epitopes.

**Results:**

Two-photon microscopy showed that the islet resident macrophages were in close contact with blood vessels and had extensive filopodial activity. Some filopodia had direct access to the vessel lumen and captured microparticles. Addition of glucose at high concentration reduced the degree of filopodia sampling of islets. This finding applied to in vivo injection of glucose or to in vitro cultures. Ultrastructural examination showed the close contacts of macrophages with beta cells. Such macrophages contained intact dense core granules. Functional studies in NOD mice indicated that the macrophages presented insulin peptides to insulin-reactive T cells. Presentation was increased after glucose challenge either ex vivo or after an in vivo pulse. In agreement with the morphological findings, presentation was not affected by insulin receptor blockade.

**Conclusions/interpretation:**

Islet resident macrophages are highly active, sampling large areas of the islets and blood contents and capturing beta cell granules. After such interactions, macrophages present immunogenic insulin to specific autoreactive T cells.

**Electronic supplementary material:**

The online version of this article (10.1007/s00125-018-4592-4) contains peer-reviewed but unedited supplementary material, which is available to authorised users.

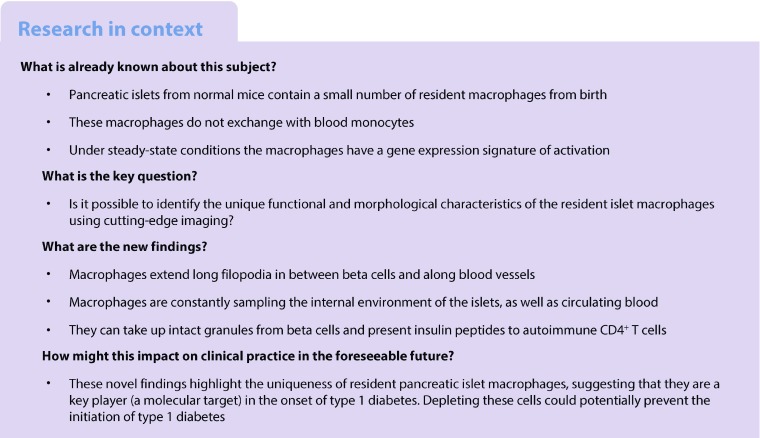



## Introduction

In all species, islets of Langerhans contain myeloid cells distributed among the endocrine cells. In the mouse, the species where most studies have been carried out, the myeloid cells are mostly represented by typical macrophages (reviewed in [1]). Early studies that identified myeloid cells in islets claimed that they were likely to be ‘passenger leucocytes’; of interest, such cells were a component of the allogeneic response to islet transplantation [2, 3]. Recent investigations examining cell surface markers and gene transcriptomes have shown that the islet myeloid cells are typical macrophages that reside in the islets and are distinct from the dendritic lineage cells (DCs) [4–6]. These islet macrophages are found from birth, replicate in the islets, and are minimally (if at all) replaced by blood monocytes [4]. Lineage-tracing studies indicate that the islet macrophages are derived from definitive haematopoiesis, while, in contrast, those residing in the inter-acinar stroma are derived from yolk sac haematopoiesis [4]. Islet macrophages are highly activated independent of any inflammation [7]. They have an M1-like profile, distinct from the M2-like profile of the stromal macrophages [4]. Recent reports have reviewed the new information on the biology of resident macrophages [8–10]. In the islet, macrophages are in intimate contact with beta cells. Through this cell contact, the beta cell–macrophage ‘synapse’, the macrophages take up insulin-containing vesicles [11]. In the context of autoimmune type 1 diabetes, these findings need to be taken into consideration. Autoimmune recognition of beta cell antigens, such as insulin, requires an intermediate cell bearing class II major histocompatibility complex (MHCII) proteins. The islet macrophages and DCs, some of which enter islets, are key cells in this process.

**Fig. 1:**
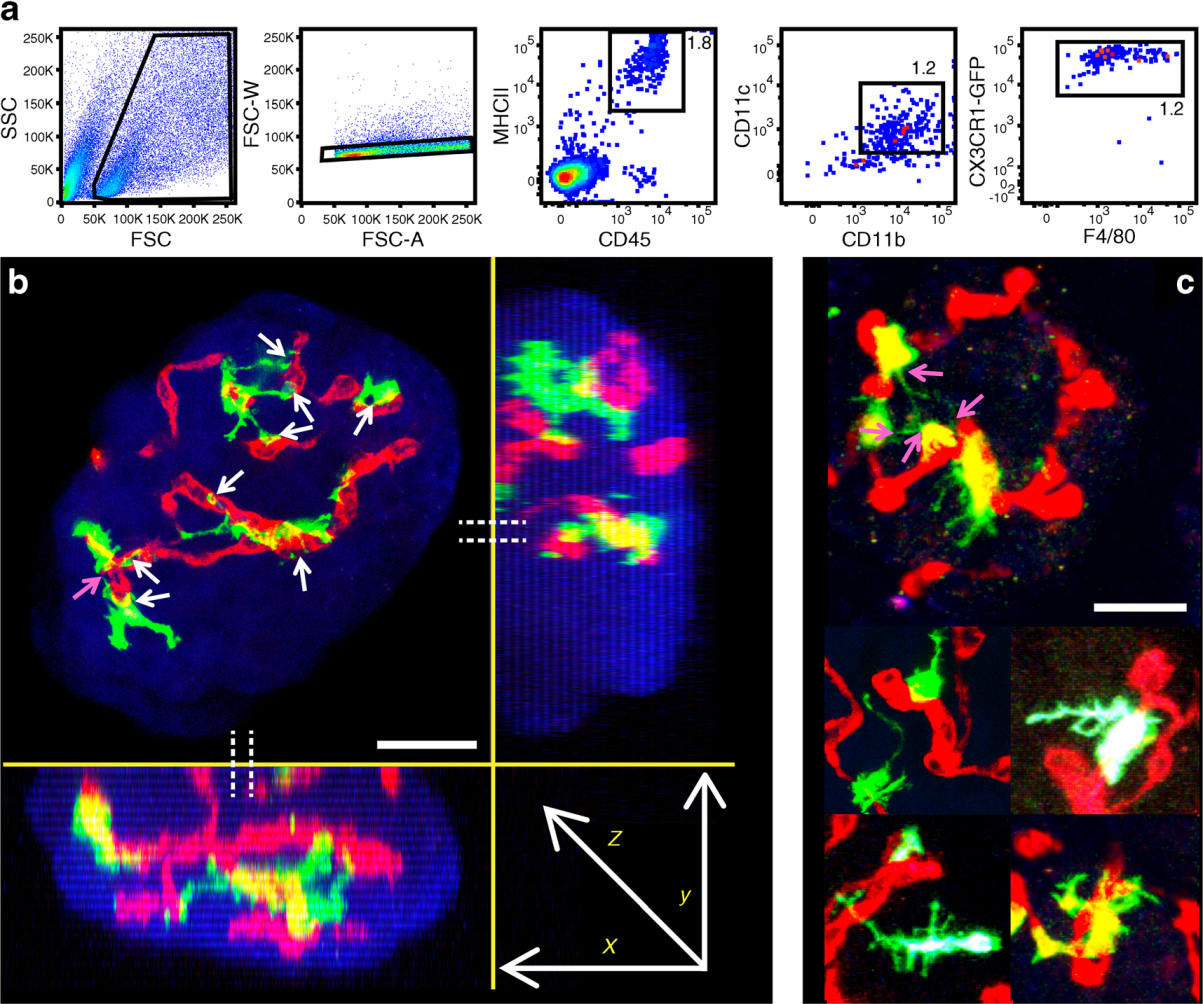
Three-dimensional two-photon microscopy of *Cx3cr1*^+/GFP^ macrophages in islets and flow cytometric analysis of the macrophages. (**a**) Flow analysis. Islet cells were isolated and labelled with antibodies directed against the indicated markers. Cells were first gated using forward-scatter (FSC) and side-scatter (SSC) followed by forward-scatter area (FSC-A) and forward-scatter width (FSC-W) to identify single cells. The CD45^+^MHCII^+^ cells were selected for analysis. The majority of these were CD11b^+^/CD11c^+^ and Cx3cr1^+/GFP^/F4/80^+^. Thus, all the CX3CR1^+^ cells were macrophages. (**b**) Three-dimensional two-photon microscopy of *Cx3cr1*^+/GFP^ macrophages in the islet. Blue, second harmonic signal and autofluorescence; green, GFP; red, vasculature. Side views show computerised optical sections (white dotted lines show location of optical sections *x*:*z* and *y*:*z*). Macrophages scan the whole islet in all directions and form filopodia to the vessel lumen (white arrows) and touch each other (pink arrow). Please compare *x*:*z* and *x*:*y* with fly-through animation in ESM Video 1. (**c**) Morphology of macrophages under steady-state conditions (pink arrows indicate interactions between macrophage filopodia). These images are representative of 12 mice; 10–20 islets imaged per mouse. Scale bars, 25 μm


In order to further extend our knowledge on the behaviour of islet macrophages, in this study we carried out live imaging of explanted islets under physiological conditions (electronic supplementary material [ESM] Fig. 1) and conducted electron microscopy studies, all in non-diabetic mice. We also performed functional assays in NOD mice, in order to correlate the morphological findings with the response of autoreactive CD4^+^ T cells to insulin peptides presented by the MHCII molecules of macrophages.

## Materials and methods

### Mice

*Cx3cr1*^+/GFP^ heterozygous mice (B6.129P-*Cx3cr1*^*tm1Litt*^/J), NOD.*Rag1*^−/−^ (NOD.129S7(B6)-*Rag1*^*tm1Mom*^/J) mice, B6.g7 mice, C57BL/6 mice and NOD mice were bred at our facilities. Information of these strains can be found at the Jackson Laboratory website (www.jax.org). Our facilities are approved by the Association for Assessment and Accreditation of Laboratory Animal Care (AAALAC). All protocols for mouse handling have been approved.

### Flow cytometry of dispersed islet cells

Islets were isolated from 6-week-old *Cx3cr1*^+/GFP^ mice (four mice in total). Islets were hand-picked and dispersed using non-enzymatic dispersion solution (Sigma, St. Louis, MO, USA) for 3 min at 37°C. Islet cells were then spun down and suspended in Mouse BD Fc Block (BD Biosciences, San Jose, CA USA). Cells were stained with the following antibodies at a final concentration of 500 ng/ml: CD11b-APC Cy7; F4/80-PerCP Cy5.5; CD11c-APC; I-A/I-E-PacificBlue and CD45-BV510 (all antibodies from BioLegend, San Diego, CA, USA). After staining with antibody in the presence of Fc block, the islet cells were examined on a BD FACSCanto II (BD Biosciences, San Jose, CA, USA) and analysed with FlowJo, version 10.0.8 (BD Biosciences).

### Islet isolation and immunological assays

Islets were isolated as described previously [4, 5] and cultured in CO_2_-independent medium (Invitrogen, Waltham, MA, USA) in the various experiments.

For immunological assays, islets from NOD.*Rag1*^−/−^ mice or from very young NOD male mice were examined. Islets were treated with either 3.8 mmol/l or 25 mmol/l glucose for 30 min to 1 h at 37°C, and then dispersed. 5 × 10^4^ islet cells were plated with 5 × 10^4^ hybridomas (IIT-3 and 8F10 are insulin-reactive hybridomas generated by us [12] that recognise insulin B:13–21 and B:12–20, respectively—see description and full details in the Results section) in a total volume of 200 μl in the wells of 96 well tissue culture plates. The cells were incubated overnight at 37°C and the IL-2 content was assayed. In the case of in vivo challenge with glucose the mice were given an intravenous injection of glucose (4 g/kg), and killed after 30 min, after which islets were isolated and tested. Testing for the effects of the insulin receptor blocker S961, the islets were isolated and incubated in 5 mmol/l or 25 mmol/l glucose in the presence or absence of 500 nmol/l S961 (Phoenix Pharmaceuticals, Burlingame, CA, USA) at 37°C. After 1 h, islets were tested for antigen presentation. In a different manipulation, an aliquot of islets were isolated and immediately dispersed. A second aliquot was pre-treated for 10 min in the absence or presence of 1 μmol/l S961 and then incubated for 1 h at 37°C in the presence of insulin, after which the islets were tested for antigen presentation. Experiments are duplicates of islets pooled from 6 to 8 mice. Data are expressed as the means±SD and an unpaired *t* test was performed.

### Electron micrographs

Islets from *Cx3cr1*^+/GFP^, B6.g7, NOD.*Rag1*^−/−^ mice and non-diabetic male NOD mice were studied. Islets were fixed in 2% (vol./vol.) paraformaldehyde/2.5% (vol./vol.) glutaraldehyde/100 mmol/l sodium cacodylate (all Sigma-Aldrich, St. Louis, MO, USA), incubated for 1 h on ice and further processed using standard techniques. Microscopy was performed using a JEOL 1200 EX transmission electron microscope (JEOL USA, Peabody, MA, USA).

### Two-photon microscopy and analysis

Islets from *Cx3cr1*^+/GFP^ mice were cultured in CO_2_-independent medium (Invitrogen, Waltham, MA, USA) at 37°C. The medium was supplemented with 10% (vol./vol.) fetal calf serum, and its basal content of glucose was 3.8 mmol/l. In some experiments, glucose was added to a final concentration of 25 mmol/l. Islets were placed between a 50 μm mesh and a quartz coverslip (Ted-Pella, Redding, CA, USA); schematic shown in ESM Fig. 2. Islets from mice intravenously injected with 50 nm Fluoresbite microparticles (Polysciences, Warrington, PA, USA) were examined in the same way.

Images were collected using a customised Leica SP8 two-photon microscope (Leica Microsystems, Wetzlar, Germany) equipped with a 25×, 0.95 NA water-immersion objective and a Mai Tai HP DeepSee Laser (Spectra-Physics, Mountain View, CA, USA) tuned to 895 nm. Fluorescence emission was guided directly to hybrid photodetectors. For signal separation, three dichroic beam splitters (Semrock, Rochester, NY, USA) were used at 458, 495 and 560 nm (FF458-Di02, FF495-Di03 and FF560-Di01). To counterstain capillaries, animals were injected with 80 μl of DyLight 594-labelled tomato lectin (Vector Laboratories, Burlingame, CA, USA) 5 min before harvesting the islets. Islets were immediately placed into a customised imaging chamber kept at physiological pH and temperature (ESM Fig. 2) and were examined for up to 2 h. Most experiments focused on the 15–30 min time period. Islets were imaged first under conditions of low glucose (3.8 mmol/l in the media), after which glucose was added to increase the concentration to 25 mmol/l. Between 15 and 25 optical sections (2.5 μm apart) were taken at regular time intervals (see ESM videos) in order to capture cell dynamics within the islet.

Image sequences were transferred to Imaris software, version 8.4.1 (Bitplane, Belfast, UK). In the case of islets examined after high glucose exposure, the data were collected after the first 15 min. Drift correction was performed, and Maximum Intensity Projections (MIPs) were generated using Imaris XTension ‘Project to 2D’. 60 × 60 μm single channel crops containing whole cells of the MIP were taken to evaluate the surveying movement of the cells. These crops were exported into Fiji (https://fiji.sc) as a stack of two-dimensional images and increased to 120 × 120 pixels so that the pixel to μm ratio was 2:1. The new images were then segmented in Matlab, version 9.2 (Natick, MA, USA) using the Matlab functions *graythresh*, which utilises Otsu’s method, minimising the intraclass variance between white and black pixels, *bwmorph* to clean the image of any isolated pixels, and then *bwareafilt* to remove any pixel clusters too small to be a cell. After segmentation, each image was subtracted from its succeeding image in the stack to determine the change over 30 s, then the changes were summed to create the heat map (equation 1). To further quantify the probing activity of the cells, a ratio of the displacement (area surveyed) was calculated relative to a reference. We chose the area of the MIP of the macrophage at the first time point as the reference. The area generated from the overlay of all 31 MIPs was divided by the area at the first time point to calculate the ratio of the probed area compared with the cell area (equation 2). To determine the percentage increase, this ratio was multiplied by 100 and then subtracted by the original 100% of the non-zero pixels of the first image (equation 3). This number allows for statistical comparisons between conditions. One drawback of this displacement ratio is that it does not explain repetitive movement, which nevertheless can be seen from the generated heat maps.

1 $$ F\left(x,y\right)={\sum}_{i=1}^n\left|{f}_{i+1}\left(x,y\right)-{f}_i\left(x,y\right)\right| $$

2 $$ DR=\frac{F\left(x,y\right)+{f}_1\left(x,y\right)}{f_1\left(x,y\right)} $$

3 $$ \% Increase=\left( DR\times 100\right)-100\% $$

*F* (*x, y*) is the accumulated movement of the images *f*_*i*_(*x, y*); *i* represents the time point for each image *f*_*i*_(*x, y*); *DR* is the displacement ratio.

### Quantification of microparticles in macrophages

The number of microparticles taken up by macrophages was identified with Imaris. Images were rendered and viewed in three dimensions. The total number of macrophages and the number of microparticle-containing macrophages per islet were counted. Results are given as percentage of microparticle-containing macrophages per islet. Statistical analysis between the groups was performed by applying a one-way ANOVA followed by Tukey’s multiple comparisons test. In order to compare the amount of microparticles taken up by macrophages over time we calculated the ratio of bead volume relative to the total macrophage volume using Imaris. The ratio is given as a percentage. Statistical analysis between the groups was performed by applying a one-way ANOVA followed by Tukey’s multiple comparisons test using GraphPad Prism, version 7.02 (GraphPad, La Jolla, CA, USA).

## Results

### In situ imaging of intact islets

Islets were isolated from C57BL/6 (B6) mice expressing green fluorescent protein under the macrophage-specific marker CX3C motif chemokine receptor 1 (CX3CR1), also known as the fractalkine receptor or G-protein coupled receptor 13 (GPR13) [13]. Such islets contain phagocytes represented by typical macrophages, identified by surface markers (Fig. 1a). Islet resident macrophages express CD11c as do DCs, which initially led them to be erroneously identified as DCs [14–17].

Video 1Three-dimensional rotation and fly-through animation through an intact islet acquired by two-photon microscopy. Mice were injected intravenously with 80 µl DyLight 594-labelled tomato lectin, islets were isolated. Second harmonic signal and autofluorescence (blue), *Cx3cr1*^+/GFP^ macrophages (green) and vasculature (red). Macrophages pervade the entire islet and several macrophage filipodia ‘anchored’ on a blood vessel (MP4 8492 kb)


Two-photon imaging revealed that the number of green fluorescent protein (GFP)-positive macrophages per islet ranged from two to 13 (*n* = 51 islets, 4.9 ± 2.6 [mean±SD]). All macrophages were found closely anchored next to blood vessels (white arrows) and were not moving freely through the islets (Fig. 1b,c). They showed continuous extensions of long, thin filopodia that derived from different points of the cell. The filopodia varied in that some were small and surrounded the macrophage body, whereas many more were long, reaching close to the edge of the islets and rapidly retracting. Those macrophages situated near the centre of the islet extended long filopodia that reached near the very edge of the islet. Some filopodia touched other macrophages (pink arrows) (Fig. 1c). The 3D imaging of the macrophages (Fig. 1b,c, ESM Fig. 1, ESM Video 1) shows the macrophages clearly pervading the whole islet and constantly probing large areas of them. The distinct contact points to the blood vasculature shows their ‘interest’ for these vessels. In essence, the macrophages sample many areas of the islet. Strikingly, some touch the edge of the blood vessels and access the vascular lumen, as will be discussed below.

**Fig. 2:**
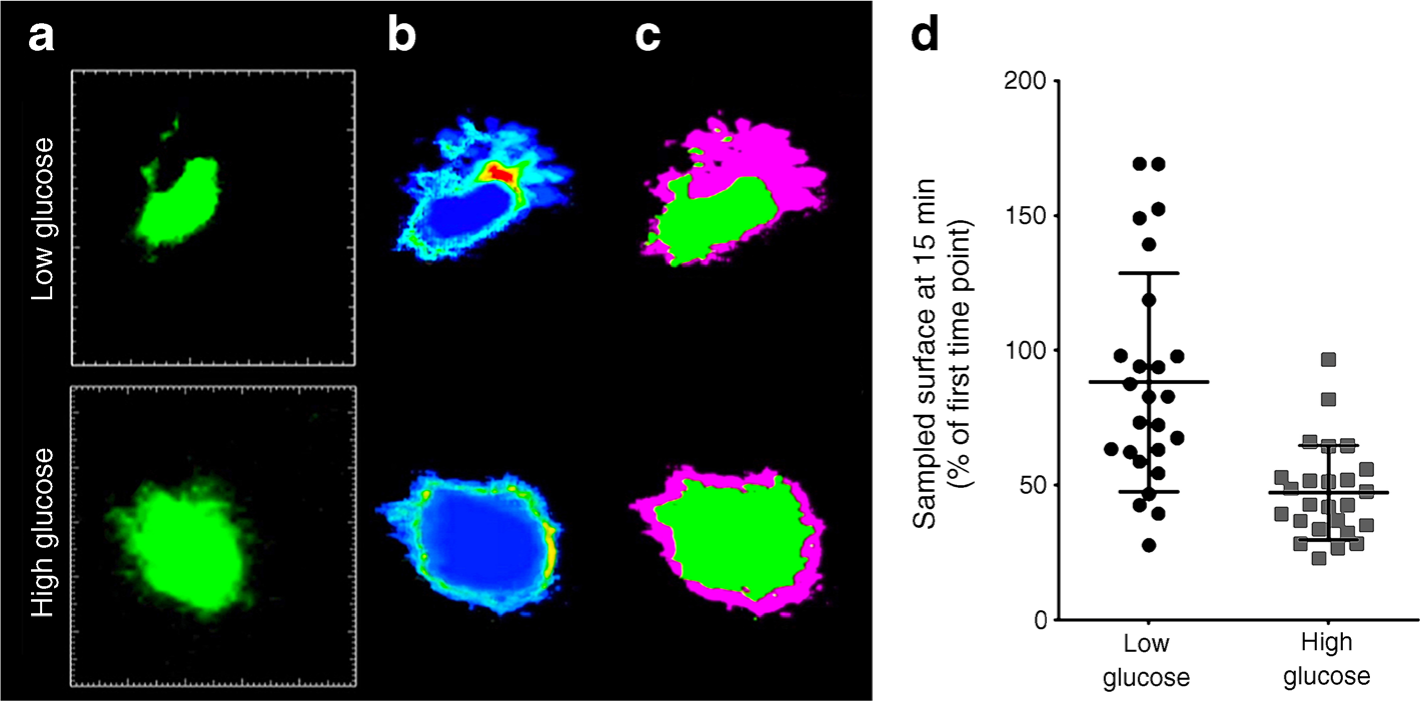
Quantitative analyses of macrophage membrane dynamics. Two-photon imaging stacks were acquired every 30 s; 31 stacks representing 15 min underwent MIP. (**a**–**c**) Representative macrophage images under a glucose concentration of 3.8 mmol/l (‘low glucose’) and a glucose concentration of 25 mmol/l (‘high glucose’) are shown. (**a**) MIP of the whole macrophage at the start of the experiment. The white 2D scale bar (60 μm × 60 μm) shows the cropped image (scale bar also applies to (**b**) and (**c**)). (**b**) The accumulated computerised MIPs from all 31 stacks over 15 min. (**c**) The computerised MIP from the first stack, in green, on top of all computerised MIPs in purple (compare with ESM Video 2). The area probed over 15 min is shown in purple. (**d**) The graph shows the surface sampled by the macrophages at the 15 min time point expressed as a percentage of the initial surface area at time 0. Each dot represents one macrophage taken from 20–40 islets. Horizontal lines indicate the mean±SD


The addition of glucose caused a change in the activity of the macrophages with thickening of the filopodia and rounding of the macrophages. We quantified these changes by a novel methodology that allowed us to quantitate the morphological changes over a given time period [18]. This method quantified filopodial activity by measuring the area covered at each time point (Fig. 2, ESM Fig. 2, ESM Video 2).

Video 2Quantitative analyses of macrophage membrane dynamics. Two-photon imaging stacks were acquired every 30 s; 31 stacks representing 15 min underwent MIP. The surface of the MIP from the first time point (green) is subtracted from the accumulated surface of all 31 frames (purple), showing the probed area over the course of 15 min. A value of 0% means there is no change from the initial shape over the course of the entire image sequence. This method can be used to quantify and compare macrophage membrane dynamics under different experimental conditions (i.e. glucose concentrations) (MOV 7694 kb)


### Macrophages capture intravenously injected microparticles

**Fig. 3:**
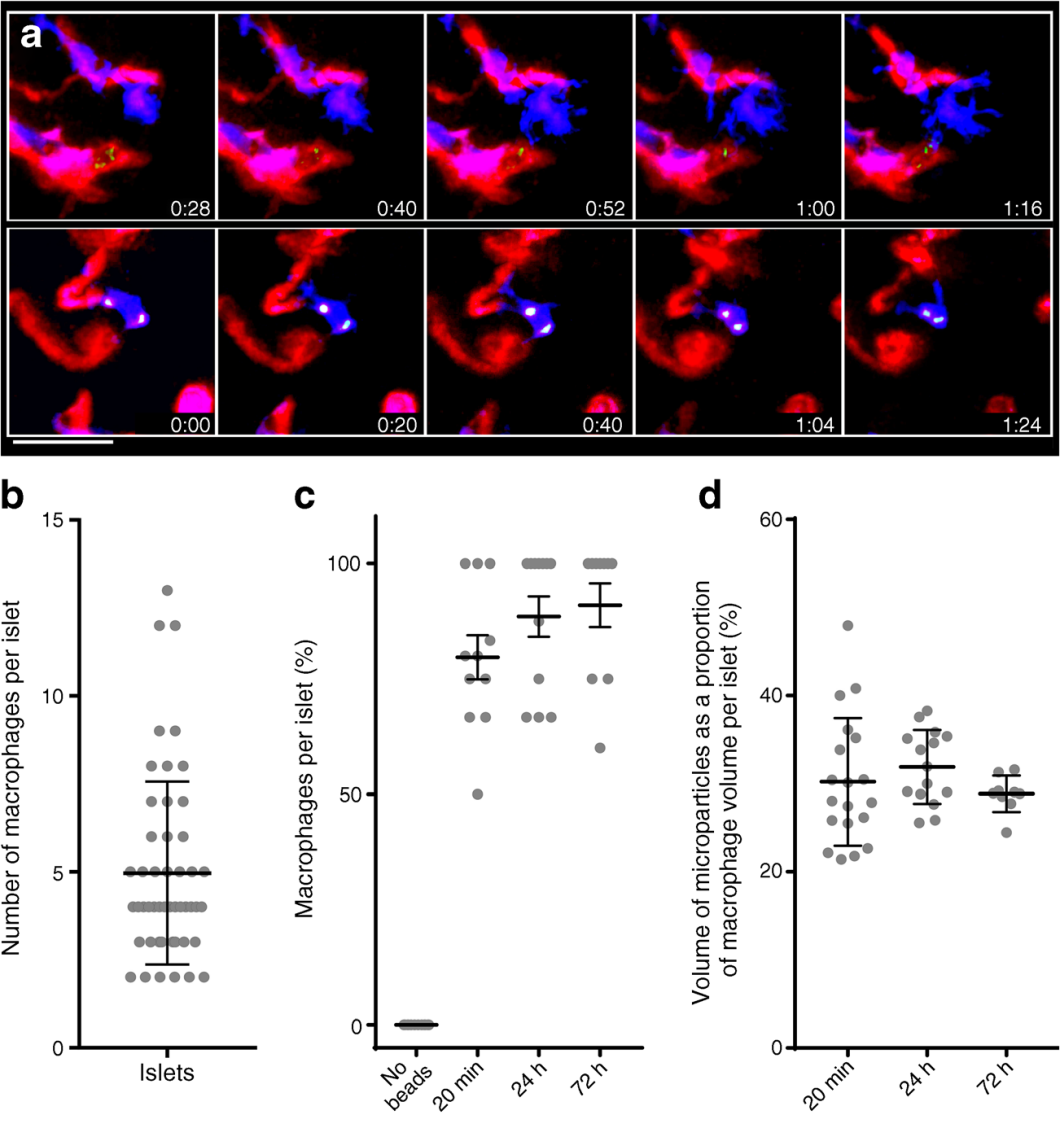
Islet macrophages capture intravascular particles. Fifty microlitres of 50 nm microparticles together with 80 μl of DyLight594-labelled tomato lectin were injected intravenously into *Cx3cr1*^+/GFP^ mice and the islets were harvested 10 min later. Islets were then placed in a customised heated chamber (as described in the Methods section and ESM Fig. 2) and examined. (**a**) The long filopodia of the macrophages (blue) extend towards the blood vessels (red) and acquire microparticles (green/white) over time. Time is given in hours:minutes. Scale bar, 25 μm. (**b**) The number of macrophages per islet (mean: 4.9). (**c**) The percentage of macrophages per islet that acquire microparticles. After 20 min, about 80% of the macrophages had taken up microparticles; there was no statistical difference in microparticle uptake at 20 min, 24 h or 72 h. (**d**) In order to semi-quantify the microparticle content per macrophage, the total 3D volume occupied by the particles was computerised. The graph shows the percentage of the macrophage volume that is occupied by microparticles. After 20 min, about 30% of the macrophage volume contained microparticles, which was similar to all other observed time points. Horizontal lines indicate the mean±SD


Because the macrophages were found in intimate contact with the blood vessel, we extended our observations by investigating whether circulating microparticles could be taken up by macrophage filopodia. To do this we injected labelled microparticles of 50 nm diameter. Such particles leave the circulation with half-time of about 5 min. Mice were killed and islets were isolated 10 min after injection. Practically all macrophages contained microparticles 10 min after injection, which was the first time period of observation. These remained associated with the macrophages when examined at the 72 h time point. A few microparticles were found inside the blood vessels, but the majority were inside the macrophages. We did not observe any microparticles free inside the islets. Strikingly, in situ two-photon time-lapse imaging showed macrophages extending filopodia into the blood vascular lumen, touching and capturing microparticles. In sum, the imaging data clearly show that the macrophages directly sample blood components with their cytoplasmic extensions (Fig. 3, ESM Video 3).

Video 3Islet macrophages capture intravascular particles. Fifty microlitres of 50 nm microparticles together with 80 μl of DyLight594-labelled tomato lectin were intravenously injected. Islets were harvested 10 min after the injection. Islets were placed in a customised heated chamber (see ESM Fig. 2) filled with CO_2_-independent medium and underwent 2P microscopy. See the CD11c^+^ macrophages (blue) stretching filopodia towards the blood vessels (red) and acquiring microparticles (green/white) over time (MP4 7400 kb)


### Ultrastructural analysis

In two initial studies we reported on electron microscope analysis of islets from non-diabetic mice in which dense core granules were identified within the macrophages [11, 15]. We have now extended these analysis testing macrophages from the *Cx3cr1*^+/GFP^ mice, as well as from two other non-diabetic mice: B6.g7 mice, i.e. C57B/6 mice expressing I-A^g7^ diabetogenic MHCII molecules, and NOD.*Rag1*^−/−^ mice, i.e. mice lacking lymphocytes. Similar results were found in islets from 4–6-week-old mice and 14-week-old mice. We also examined non-diabetic male NOD mice at the 3–6-week period, before any overt inflammation. The results were the same in all four groups of mice.

**Fig. 4:**
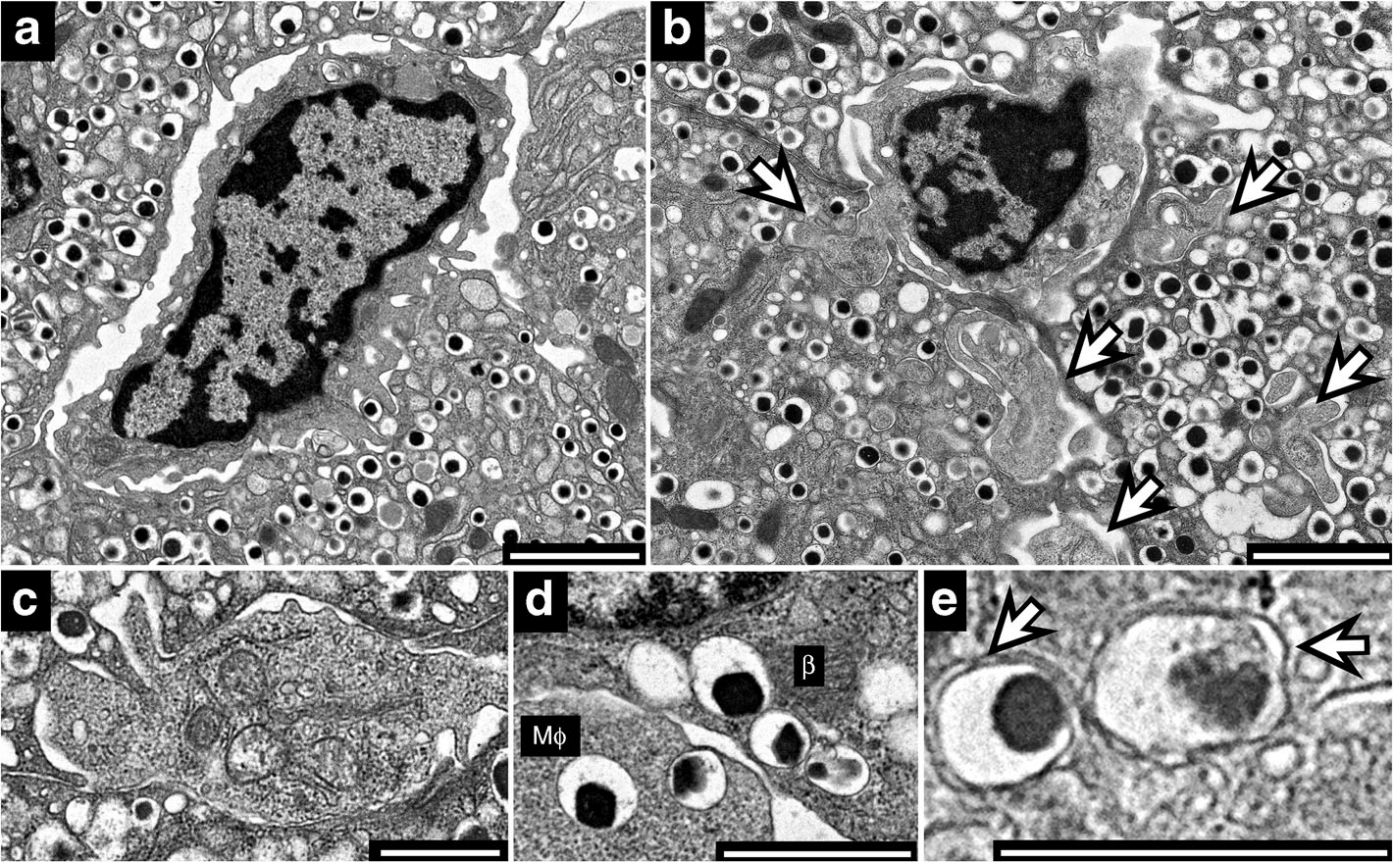
Electron microscopy of macrophages in islets of *Cx3cr1*^+/GFP^ mice. (**a**) Macrophage in close contact with beta cells. (**b**) The macrophages extend some of their filopodia into the beta cells (arrows). (**c**) High magnification of section through a macrophage filipodia in contact with insulin granules. (**d**) The macrophages take up intact insulin granules with the typical dense core and surrounding halo. β, beta cell; MΦ, macrophage. (**e**) Capsule of the dense core granule (arrows). (**a**, **b**) Scale bar, 2 μm; (**c**, **d**, **e**) scale bar, 1 μm. These images are representative of electron micrographs of islets isolated from 24 mice (2–10 islets per mouse)

The studies showed the macrophages next to blood vessels, with thin extensions extending along part of the vessel wall. The macrophages were in close contact with beta cells, i.e. in a beta cell–macrophage synapse, in which there were extensive filopodia in the area of contact. All macrophages contained vesicles of different composition. Notably, many vesicles were typical insulin-containing granules, with their characteristic dense core and surrounding halo and vesicle membrane, indicating that the vesicles had been taken up intact by the macrophages (Fig. 4). Some vesicles contained amorphous material different from the typical dense core granule. Other electron microscopy images showed thin macrophage extensions that penetrate into the beta cell. There was no indication of cell death nor have we documented free vesicles in the space between the macrophages and beta cells.

### Immunological assays

We next examined whether the addition of blood glucose to the islets ex vivo as carried out in the imaging studies had immunological relevance, i.e. whether there was an increase in the presentation of insulin peptides. Despite several attempts, we were not able to raise T cells to insulin peptides in B6 mice. Insulin is poorly auto-immunogenic in the B6 strain, a diabetic-resistant strain. Instead, islets from NOD mice were examined. We examined islets from NOD.*Rag1*^−/−^ mice, which do not develop diabetes, or from very young NOD male mice, i.e. islets from NOD mice in a non-diabetic condition. The major myeloid cell in both of these is the F4/80-positive macrophage. Both NOD and NOD.*Rag1*^−/−^ mice show macrophages from birth with identical features to those described in the B6 mouse [4, 5].

We tested the response of two sets of insulin-reactive T cells previously characterised by us [12, 19]. Those reactive to B:13–21 recognise this insulin peptide after processing of insulin, while the T cells reactive to B:12–20 recognise insulin peptides or unfolded insulin B chain. The approach was similar to the one used in the imaging analysis, i.e. a brief incubation in low or high concentrations of glucose, after which the islets were dispersed and tested against CD4^+^ T cells to insulin epitopes. We also tested the effects of a single intravenous bolus of glucose.

**Fig. 5:**
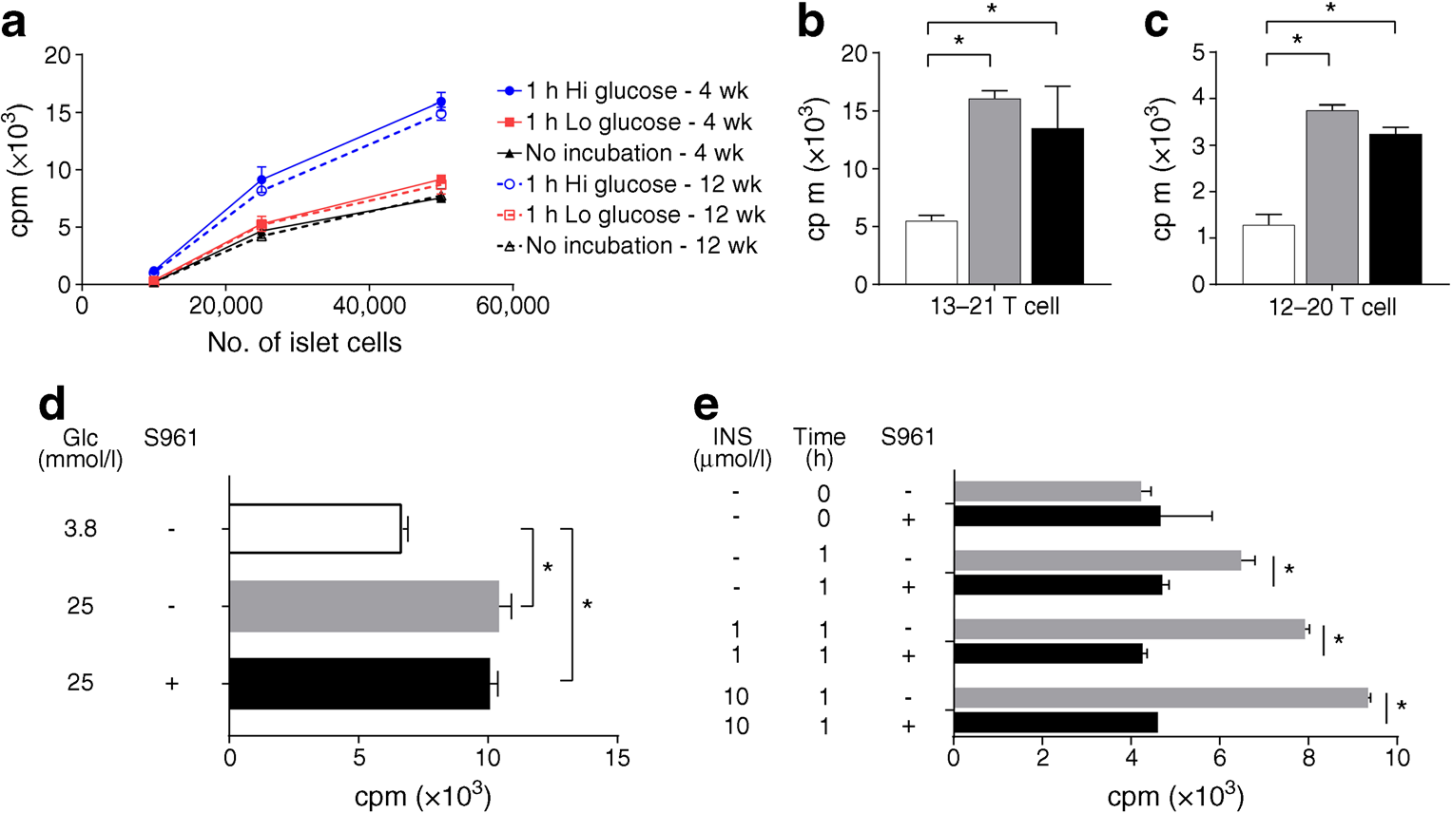
Presentation of insulin peptides by islets. (**a**) Islets were isolated from NOD.*Rag1*^–/–^ mice at either 4 or 12 weeks of age. A portion of the islet cells were dispersed immediately after harvesting (labelled ‘no incubation’), another set was dispersed after 1 h incubation with 3.8 mmol/l glucose (‘Lo glucose’) and a third after 1 h incubation with 25 mmol/l glucose (‘Hi glucose’). Each set was dispersed and then different numbers of cells were incubated with IIT-3 T cells (which react with the B:13–21 peptide segment of the insulin B chain). Incubation with high glucose led to an increase in presentation of insulin peptides. Wk, week. (**b, c**) Islets of 13-week-old male B6.g7 mice were tested against both IIT-3 T cells directed to B:13–21-(13–21 T cell) or B:12–20-(12–20 T cell). The islets were tested 30 min after intravenous injection of glucose (4 g/kg, black bars) or after 1 h ex vivo exposure to 3.8 mmol/l (white bars) or 25 mmol/l glucose (grey bars). Intravenous challenge also led to an increase in presentation of insulin peptides. Shown is a composite of two experiments with similar results. (**d**) Islets were treated as in (**a**), but one set was also treated in the presence or absence of 500 nmol/l of the insulin receptor blocker S961. S961 did not inhibit the presentation of insulin peptides induced by glucose challenge. Results were pooled from two experiments. (**e**) The experiment tested the effects of S961 in islets not challenged with glucose, which favours granule uptake (**d**). An aliquot of islets was immediately dispersed (time 0) in the presence or absence of 1 μmol/l of S961. It shows the basal level of insulin peptide presentation. This presentation was not affected by S961. The remaining islets were incubated for 1 h without or with added insulin (INS) at concentrations indicated and without or with 1 μmol/l of S961. All islets were tested againts IIT-3 T cells. Incubation for 1 h increased the basal presentation, probably due to the release of free insulin. Incubation with added insulin concentrations led to further increase in insulin peptide presentation. S961 treatment inhibited presentation of insulin to the level found at time 0. Results were pooled from duplicate experiments in 6–8 mice. Data are mean ± SD; *p < 0.05

T cells to insulin were activated by culturing them in islets dispersed immediately after harvesting. We took this as an indication that there is a basal level of uptake of the antigenic material. Approximately the same response was found in islets after culturing for 1 h in a low glucose concentration. However, the addition of glucose resulted in a marked increase as shown in Fig. 5a–c. In the first 12 experiments testing the T cells reactive to the B:13–21 peptide, the mean increase after the glucose challenge was 118% (SD: ±57%); in nine experiments, the reactivity to B:12–20 was lower than to B:13–21, but the increase on the addition of glucose was evident: a mean increase of 213% (SD: ±167). Importantly, from the perspective of the biology of presentation during diabetogenesis, the intravenous injection of glucose also led to an increase in presentation: mice were injected and killed 15 min later, islets were dispersed and tested against both T cells. In brief, a glucose challenge, which is readily sensed by beta cells, resulting in increased granule exocytosis, also results in the uptake of such granules by the resident macrophages, producing an increase in the presentation of insulin epitopes (Fig. 5). In a recent study we deleted the islet macrophages in NOD mice by administration of a monoclonal antibody to the colony-stimulating factor 1 (CSF-1) receptor. This treatment resulted in a complete absence of antigen presentation from the islets and a marked reduction in diabetes incidence [20]. (It should be noted that glucose per se has no effect on antigen presentation dynamics, as has been tested in different presenting cells, including peritoneal macrophages pulsed with insulin peptides, as shown in ESM Fig. 3).

We also evaluated whether the insulin that was presented derived from uptake of soluble insulin. Islets were cultured with 25 mmol/l glucose in the presence of S961, a peptide that blocks the insulin receptor [21], then dispersed and tested against the insulin-reactive T cells. Insulin presentation was identical to that in islets not treated with the S961 peptide (Fig. 5d). In control tests, islets cultured with S961 and then pulsed with insulin resulted in a complete reduction of presentation (Fig. 5e). (Figs 5d and 5e refer to the increase in response after the glucose challenge. There is always a basal level of presentation by the macrophages cultured with the basal level of glucose.) This last finding complements the ultrastructural examination, where the granule was identified with its own capsule inside an endosome, indicating that the uptake of granules did not result from receptor-mediated uptake of insulin.

## Discussion

The microscopical findings on islet macrophages integrate and explain a number of functional analyses. Such resident macrophages were in close contact with beta cells and showed extensive membrane activity that resulted in the uptake of intact secretory granules. It should be noted that in most endocrine organs there are close contacts of resident macrophages with the hormone-producing cell, similar to our findings in islets. A notable example is in the testes, where there are intimate interactions between Leydig cells and macrophages [22].

These changes exemplify the morphological plasticity of the macrophages. Traditionally, filopodia were not identified as a property of macrophages, but rather as being typical of DCs. However, this view has to be changed with respect to the various tissue resident macrophages. Many tissue resident macrophages extend many long filopodias (for three examples see [23–25]). Such filopodial activity may be a response to environmental cues. Many of the initial studies on macrophage morphology were performed on cultures of peritoneal macrophages or macrophages activated by interferons and these showed their round or flat appearance. The epithelioid cells of the tuberculous granuloma represent an in vivo counterpart.

We also found that the macrophages were next to blood vessels and showed no displacement from their anchored site. A previous study of ours had indicated that macrophages were in contact with blood vessels. Phase contrast studies performed by Calderon et al in our group showed thin extensions into blood vessel lumens [26]. In these studies, 500 nm latex particles coated with antibodies to MHCII proteins were injected into NOD or B6 mice. In islets isolated shortly thereafter, the beads adhered to the blood vessels but always next to a macrophage [26]. This interaction was specific in that the beads coated with anti-I-A^g7^ were found in NOD islets but beads coated with anti-I-A^b^ were not, and vice versa in the B6 mouse. The present results add to these early findings, making the important point that the intraluminal filopodia serve as the capturing instrument of luminal material. It is likely that the uptake of luminal material will be related to blood flow and variables that influence it; issues to bear in mind as we relate these findings to functional analyses.

The uptake of intact granules by the macrophages was surprising. This form of uptake is in striking contrast to the canonical mechanism of granule release from the beta cell following glucose stimulation, in which the granule membrane is incorporated into the plasma membrane. A similar phenomenon has been documented for the transfer of melanophores from melanocytes to keratinocytes (reviewed in [27]). The granules were not released from dead beta cells; the beta cells next to the macrophages appeared to be normal. It may be that the intact granules are rapidly released at the very intimate contact zone between the two cells. We should note that the uptake of granules correlated with the transfer of immunogenic insulin in the case of the diabetes-prone islets of the NOD mouse. Importantly, it was not inhibited by blocking the insulin receptor, an indication that the transfer did not involve free insulin.

We recently reported on the complex transcriptome of macrophages with a high state of activation, likely reflecting the various stimuli to which they are subjected [7]. The nature of the molecule(s) responsible for such an activation state is one key issue at present. A major source is likely to be the insulin-containing granule, with its series of bioactive molecules. Indeed, in the present studies, addition of glucose resulted in a change in morphology, with more rounding of the filopodia, an indication that the cell was likely to be responding to some product of the granule, such as ATP or insulin. We have also previously reported that the islet macrophages can sense bioactive products from the circulation [7]. Our results here on the uptake of microparticles by the macrophages through extensions into the blood lumen is another major proof of this sensing role.

The function of the activation state and of the continuous sensing of beta cells as reported here need explanation. We speculate that the macrophage must have a protective role against blood borne inflammatory moieties. Finally, the macrophage, with its capacity to take up beta cell granules and its activation state, should influence the development and progression of autoimmune diabetes in conditions in which the genetic program favours it. This is the case in the NOD mouse, in which eliminating the macrophages results in a marked reduction in diabetes incidence [20]. In the NOD mouse, another phagocyte makes a seminal contribution to diabetogenesis, namely, the XCR1^+^ DC, which appears in islets at the time that T cells enter, at about 3–4 weeks of age, but then increases [5]. How the two phagocytes interact to bring about the autoimmune program is the subject of studies at present. Finally, the macrophage–beta cell interaction is a truly symbiotic one. That macrophages influence the development of the islet is well documented in studies from mice lacking macrophages by genetic mutation of CSF-1: their islets poorly develop and have reduced cellular mass [4, 15, 28].

## Electronic supplementary material

ESM (PDF 416 kb)

Video 1 Three-dimensional rotation and fly-through animation through an intact islet acquired by two-photon microscopy. Mice were injected intravenously with 80 µl DyLight 594-labelled tomato lectin, islets were isolated. Second harmonic signal and autofluorescence (blue), *Cx3cr1*^+/GFP^ macrophages (green) and vasculature (red). Macrophages pervade the entire islet and several macrophage filipodia ‘anchored’ on a blood vessel (MP4 8492 kb)

Video 2 Quantitative analyses of macrophage membrane dynamics. Two-photon imaging stacks were acquired every 30 s; 31 stacks representing 15 min underwent MIP. The surface of the MIP from the first time point (green) is subtracted from the accumulated surface of all 31 frames (purple), showing the probed area over the course of 15 min. A value of 0% means there is no change from the initial shape over the course of the entire image sequence. This method can be used to quantify and compare macrophage membrane dynamics under different experimental conditions (i.e. glucose concentrations) (MOV 7694 kb)

Video 3 Islet macrophages capture intravascular particles. Fifty microlitres of 50 nm microparticles together with 80 μl of DyLight594-labelled tomato lectin were intravenously injected. Islets were harvested 10 min after the injection. Islets were placed in a customised heated chamber (see ESM Fig. 2) filled with CO_2_-independent medium and underwent 2P microscopy. See the CD11c^+^ macrophages (blue) stretching filopodia towards the blood vessels (red) and acquiring microparticles (green/white) over time (MP4 7400 kb)

## Abbreviations

CSF-1: Colony-stimulating factor 1
CX3CR1: CX3C motif chemokine receptor 1
DC: Dendritic cell
GFP: Green fluorescent protein
MHCII: Major histocompatibility complex class II
MIP: Maximum Intensity Projection
