# Supplementary material for: The resident macrophages in murine pancreatic islets are constantly probing their local environment, capturing beta cell granules and blood particles
Source: Diabetologia. 2018 Mar 27;61(6):1374–83. doi: 10.1007/s00125-018-4592-4 (PMC5938291; doi:10.1007/s00125-018-4592-4)
Supplement: Supplementary file 4 — (PDF 416 kb) [file 125_2018_4592_MOESM1_ESM.pdf]

**ESM Fig. 1**

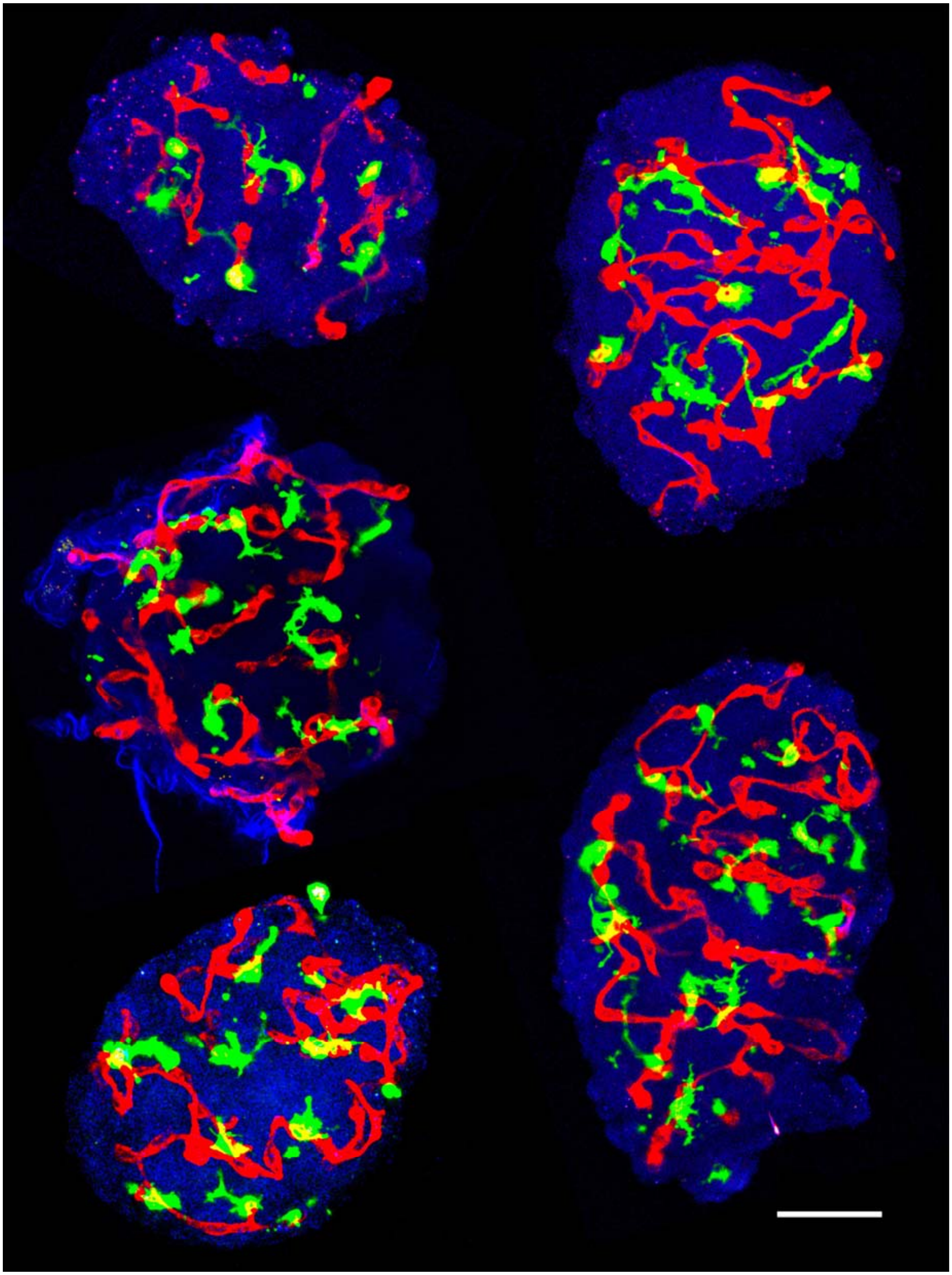

Electronic Supplemental Figure 1: Further images of the 3-Dimensional two-photon microscopy of *Cx3cr1*$^{+/GFP}$ islet macrophages. Mice were injected intravenous with 80µg DyLight 594 labeled Tomato Lectin and islets were harvested and fixed in paraformaldehyde: second harmonic signal and auto fluorescence (blue), GFP (green) and vasculature (red). The figure shows Maximum Intensity Projections of all z-stacks of 5 representative islets from different experiments and have been merged into one figure. Scale bar 50µm.

**ESM Fig. 2**

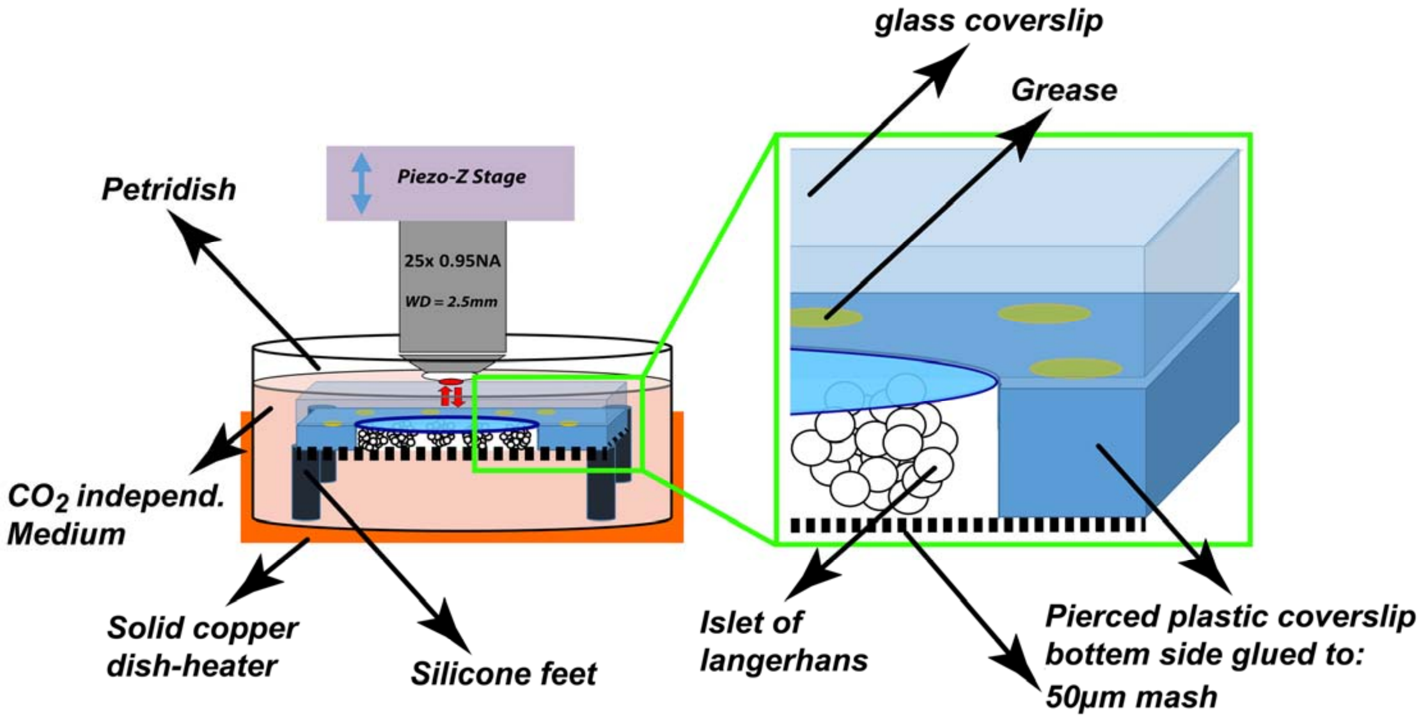


Electronic Supplemental Figure 2: Schematic diagram of preparation for *in situ* two- photon imaging of intact islets. Isolated islets were placed in the pocket between plastic cover slip which was sealed with a 50µm mash on the bottom to allow interchange with the media; the preparation was covered with a glass coverslip. This setup fits into a 60mm Petri dish in an in-house developed copper heating plate for regulating the temperature.

**ESM Fig. 3**

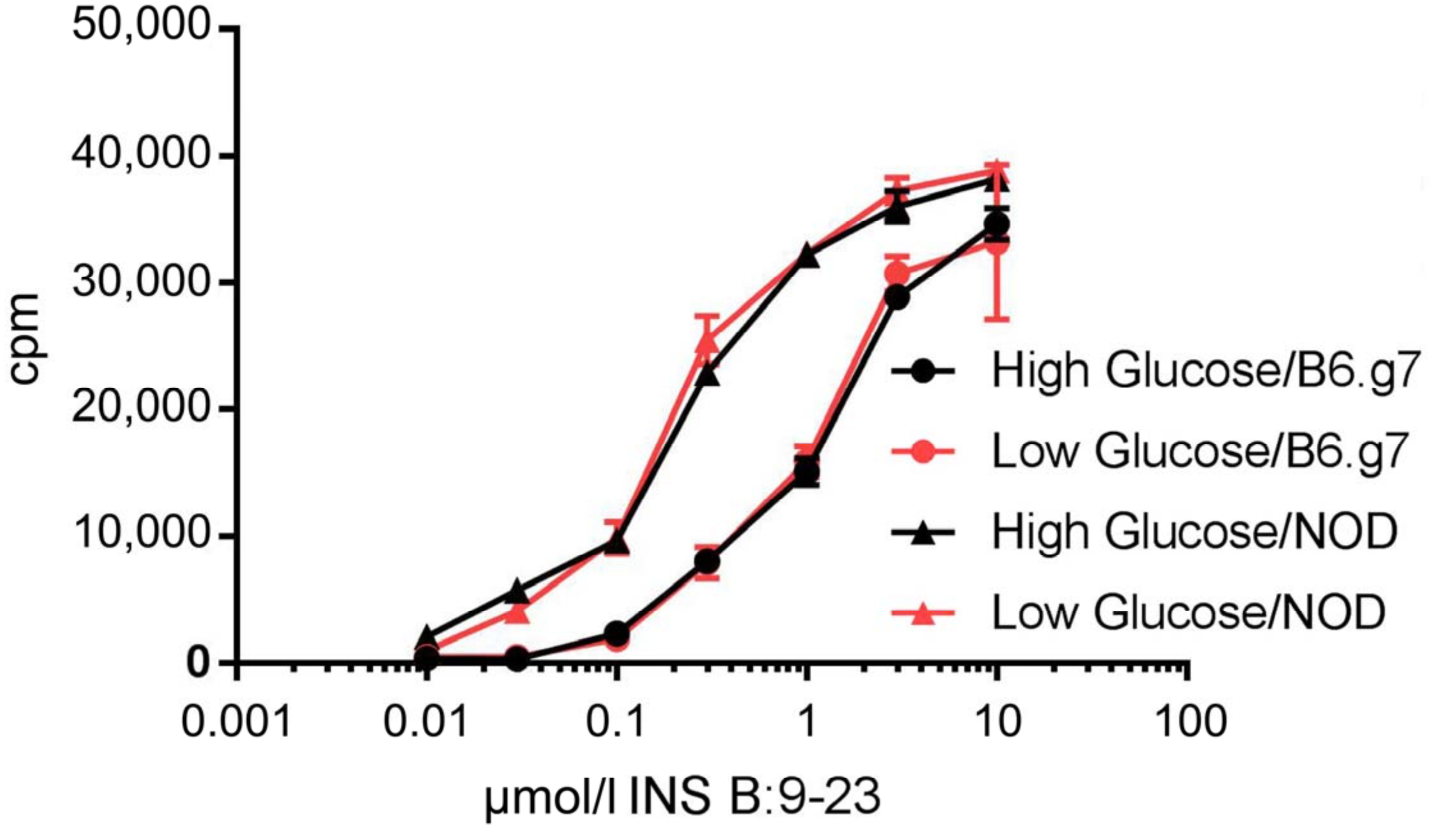


Electronic Supplemental Figure 3: Levels of glucose have no effect on insulin presentation by macrophages. Macrophages were harvested from the peritoneal cavity of mice three days after the local injection of 200ug of Concanavalin A. This results in an influx of macrophages expressing high level of MHC-II. Macrophages were cultured with 5 or 25 mmol/L of glucose in the presence of different concentrations of the insulin peptide B:9-23 and tested against an insulin reactive CD4 T cells directed to the B chain segment 13-21. Macrophages were harvested from male mice, 8-10 weeks of age of either the B6.g7 or NOD strain. The levels of presentation was not affected by glucose levels.

## ESM Video legends

**ESM Video 1** Three-dimensional rotation and fly-through animation through an intact islet acquired by two-photon microscopy. Mice were injected intravenously with 80 µl DyLight 594-labelled tomato lectin, islets were isolated. Second harmonic signal and autofluorescence (blue), $\mathit{Cx3cr1}^{+/GFP}$ macrophages (green) and vasculature (red). Macrophages pervade the entire islet and several macrophage filipodia ‘anchored’ on a blood vessel

**ESM Video 2** Quantitative analyses of macrophage membrane dynamics. Two-photon imaging stacks were acquired every 30 s; 31 stacks representing 15 min underwent MIP. The surface of the MIP from the first time point (green) is subtracted from the accumulated surface of all 31 frames (purple), showing the probed area over the course of 15 min. A value of 0% means there is no change from the initial shape over the course of the entire image sequence. This method can be used to quantify and compare macrophage membrane dynamics under different experimental conditions (i.e. glucose concentrations)

**ESM Video 3** Islet macrophages capture intravascular particles. Fifty microlitres of 50 nm microparticles together with 80 µl of DyLight594-labelled tomato lectin were intravenously injected. Islets were harvested 10 min after the injection. Islets were placed in a customised heated chamber (see ESM Fig. 2) filled with $CO_2$-independent medium and underwent 2P microscopy. See the $CD11c^{+}$ macrophages (blue) stretching filopodia towards the blood vessels (red) and acquiring microparticles (green/white) over time
